# Myelolipoma of the posterior mediastinum: report of a case

**DOI:** 10.1007/s11748-013-0230-8

**Published:** 2013-03-11

**Authors:** Toshinari Ema, Ryoji Kawano

**Affiliations:** Department of Thoracic Surgery, Itabashi Central Medical Center, 2-12-7 Azusawa, Itabashi-ku, Tokyo, 174-0051 Japan

**Keywords:** Extraadorenal myelolipoma, Mediastinal tumor, Myelolipoma

## Abstract

Myelolipoma is an uncommon tumor composed of adipose tissue and normal hematopoietic elements, and is most often found in the adrenal glands. We report a patient with a posterior mediastinal myelolipoma. The 68-year-old male patient showed a right lower mediastinal shadow in a chest X-ray. A computed tomographic scan demonstrated a right posterior mediastinal mass. Magnetic resonance imaging provided additional useful information. The patient underwent a successful resection under video-assisted thoracoscopic surgery.

## Introduction

Mediastinal extraadrenal myelolipoma is a very rare tumor composed of fat cells and mature bone marrow cells including normoblastic, granulocytic and megakaryocytic series. We can find only a few cases of extraadrenal myelolipoma located in thoracic cavity. We report a case of myelolipoma originating in the posterior mediastinum.

## Case

**Fig. 1:**
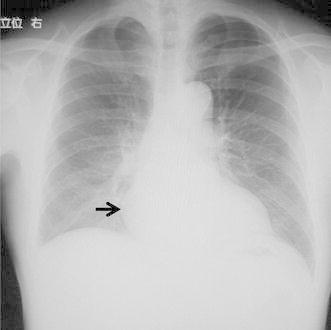
Chest radiography shows a mass on the right lower mediastinum

**Fig. 2:**
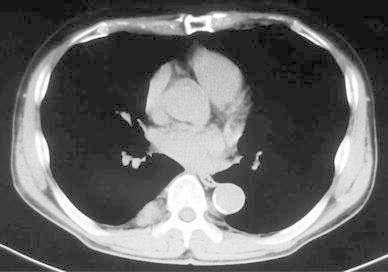
Chest CT shows a tumor located in the right posterior mediastinum. It is 2.5 cm in diameter beside the T8 and T9 thoracic vertebrae

**Fig. 3:**
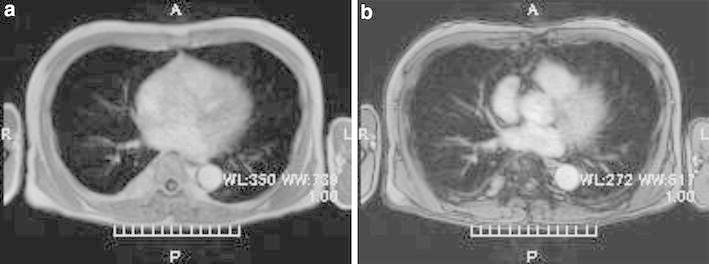
Magnetic resonance imaging (MRI). According to T1 enhanced dual image at (TR165, TE4.8), the tumor is revealed as high intensity (Fig. 3a), however, at (TR165, TE2.3) it is revealed as partially low intensity (Fig. 3b). It suggests that the tumor contains fat component

A 68-year-old man was referred to the Itabashi Central Medical Center for a surgical resection of posterior mediastinal tumor in November 2011. He was pointed out with a mediastinal mass by a health checkup. Previous history of the patient was not significant. The result of physical examination was within normal limits. Chest radiography showed a mass on the right lower mediastinum (Fig. 1). Chest CT showed a tumor located in the right posterior mediastinum. It was 2.5 cm in diameter beside the T8 and T9 thoracic vertebrae (Fig. 2). According to T1 enhanced dual image at (TR165, TE4.8) of magnetic resonance imaging (MRI), the tumor is revealed as high intensity (Fig. 3a); however, at (TR165, TE2.3) it is revealed as partially low intensity (Fig. 3b). It suggests that the tumor contains fat component.

**Fig. 4:**
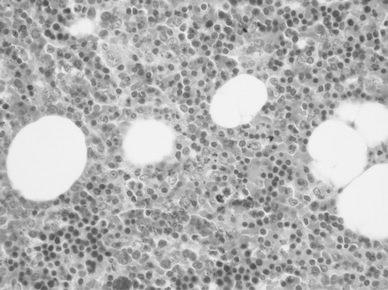
A microscopic examination reveals predominant mature adipose tissue with hematopoietic tissue

We performed a tumor resection under video-assisted thoracoscopic surgery. The tumor was reddish brown and covered with parietal pleura. It was soft and encapsulated, appearing as hemangioma. The patient was discharged with no complications on the 7th operative day, and thereafter followed up as outpatient. In the macroscopic findings, size of the specimen was 3.0 × 2.7 × 2.5 cm, and was well circumscribed and encapsulated. A microscopic examination revealed a predominant mature adipose tissue with hematopoietic tissue (Fig. 4). The final diagnosis was mediastinal extra adrenal myelolipoma.

## Discussion

Myelolipoma was first described by Gierke in 1905 and the term “myelolipoma” was also used by Oberling in 1929 [1, 2]. Extra adrenal myelolipoma was first reported by Krag in 1972 [3].

Myelolipoma is a benign nonfunctional tumor composed of extensive hematopoietic tissue and sparse fatty tissue, and is most often found in the adrenal grand. It mostly occurs to a unilateral adrenal cortex, but it is sometimes found in the anterior surface of sacrum and retroperitoneum around the kidney out of the adrenal gland. Although there are reported cases of the tumor located in stomach, liver, lungs, and mediastinum, they are all very rare [4]. The report of mediastinum such as this case is approximately 3 % of the whole myelolipoma, mostly in the lower posterior mediastinum vertebra [5, 6].

Theories on the origin of myelolipoma include the followings:

1. Ectopic hematopoietic stem cells originate and differentiate into myelolipoma.
2. Mesenchyma system cells of the ectopic adrenal tissue differentiate into myelolipoma [7, 8].

Histologically, in case of myelolipoma, some blood-forming tissues are found in abundant adipose tissue where erythroblast pro-hyperplasia is not present, and the accumulation of lymphocyte is often present [9].

CT and MRI are useful for diagnosis. CT shows the mass shadow that demonstrates a smooth and clear border and a localized low density area, whereas MRI shows high intensity regions on both T1, and T2-weighted image. In addition, by comparing original MRI image and fat suppression image, it is able to find fat-consisting tumor. These findings are helpful in diagnosis [10]. In this case, T1 enhanced dual image is useful for diagnose.

Most of the patients having this tumor are asymptomatic and often detected incidentally by chest X-ray examination. If pressure symptom or intratumoral bleeding is not seen, the patient is put to follow-up.

However, it is very difficult to definitively diagnose a tumor occurring in the thoracic area. In case of the report, it was suspected as a neurogenic tumor before the operation. Other differential diagnoses include a bronchogenic tumor, a malignant lymphoma, a lipoma, and a blood-forming organ tumor out of marrow. Since it is difficult to distinguish among them before operation, eventually we are to depend on histological diagnosis.

Considering that other available methods such as aspiration cytology or CT-guided biopsy can be as invasive, video-assisted thoracoscopic surgery may be better as means of both diagnosis and treatment.

## Conclusion

We experienced the case of the myelolipoma in mediastinum. Although primary myelolipoma is very rare, knowledge of this tumor is important for the differential diagnosis of mediastinal tumor.

## References

[1] Gierke E (1905). Uber Knochenmarksgwebe in der Nebenniere. Zeiglers Beitr Pathol Ant.

[2] Oberling C (1929). Les myelolipomamateuses. Bull Assoc Fr Etudes. Cancer.

[3] Krag D, Reich SB (1972). Heterotopic bone mallow (myelolipoma) of the mediastinum. Chest.

[4] Tamura K, Taniguchi H (2010). Myelolipoma of the posterior mediastinum. Jpn J Thorac Surg.

[5] Franiel T, Fleischer B, Raab BW, Fuzesi L (2004). Bilateral thoracic extraadorenal myelolipoma. Eur J Cardiothorac Surg.

[6] Koizumi J, Harada H, Yamamoto N, Shihisa N, Ogasa T, Takahashi M (1999). A case of mediastinal myelolipoma. The Jpn J Thorac Surg.

[7] Spanra R, Saleh HA, Khatib G (1999). Fine needle aspiration diagnosis of extraadrenal myelolipoma presenting as a pleural mass. A case report. Acta Cytol.

[8] Minamiy Y, Abo S, Kitamura M, Izumi K (1997). Mediastinal extraadrenal myelolipoma: report of a case. Surg Today.

[9] Rao P, Kenney PJ, Wanger BJ, Davidson AJ (1997). Imaging and pathologic features of myelolipoma. Radiographics.

[10] Kim K, Koo BC, Davis JT, Franco-Saenz R (1984). Primary myelolipoma of mediastinum. J Comput Tomogr.

